# Differential Expression of miRNAs in the Respiratory Tree of the Sea Cucumber *Apostichopus japonicus* Under Hypoxia Stress

**DOI:** 10.1534/g3.117.1129

**Published:** 2017-09-15

**Authors:** Da Huo, Lina Sun, Xiaoni Li, Xiaoshang Ru, Shilin Liu, Libin Zhang, Lili Xing, Hongsheng Yang

**Affiliations:** * Chinese Academy of Sciences Key Laboratory of Marine Ecology and Environmental Sciences, Institute of Oceanology, Chinese Academy of Sciences, Qingdao 266071, China; † Laboratory for Marine Ecology and Environmental Science, Qingdao National Laboratory for Marine Science and Technology, Qingdao 266071, China; ‡ University of Chinese Academy of Sciences, Beijing 100049, China

**Keywords:** miRNA, sea cucumber, hypoxia stress, respiratory tree, Illumina deep sequencing

## Abstract

The sea cucumber, an important economic species, has encountered high mortality since 2013 in northern China because of seasonal environmental stress such as hypoxia, high temperature, and low salinity. MicroRNAs (miRNAs) are important in regulating gene expression in marine organisms in response to environmental change. In this study, high-throughput sequencing was used to investigate alterations in miRNA expression in the sea cucumber under different levels of dissolved oxygen (DO). Nine small RNA libraries were constructed from the sea cucumber respiratory trees. A total of 26 differentially expressed miRNAs, including 12 upregulated and 14 downregulated miRNAs, were observed in severe hypoxia (DO 2 mg/L) compared with mild hypoxia (DO 4 mg/L) and normoxic conditions (DO 8 mg/L). Twelve differentially expressed miRNAs were clustered in severe hypoxia. In addition, real-time PCR revealed that 14 randomly selected differentially expressed miRNAs showed significantly increased expressions in severe hypoxia and the expressions of nine miRNAs, including key miRNAs such as Aja-miR-1, Aja-miR-2008, and Aja-miR-184, were consistent with the sequencing results. Moreover, gene ontology and pathway analyses of putative target genes suggest that these miRNAs are important in redox, transport, transcription, and hydrolysis under hypoxia stress. Notably, novel-miR-1, novel-miR-2, and novel-miR-3 were specifically clustered and upregulated in severe hypoxia, which may provide new insights into novel “hypoxamiR” identification. These results will provide a basis for future studies of miRNA regulation and molecular adaptive mechanisms in sea cucumbers under hypoxia stress.

Sea cucumber (*Apostichopus japonicus*), which has a high nutritional and pharmaceutical value, is an important economic species that is widely cultured in East Asia, and in China, 90,000 tons (live weight) are harvested every year (Chang and Song 2004; Wang *et al.* 2005, 2015). Suitable environmental factors, such as temperature, salinity, and dissolved oxygen (DO), are important in the aquaculture of sea cucumber. Previous research has illustrated that the DO level should be maintained over 4 mg/L in agriculture ponds (Song 2009). However, since 2013, sea cucumbers have encountered huge mortality in summer because of hypoxia, which is often defined as DO levels below 2 mg/L in aquatic systems (Huang *et al.* 2012; Liu 2014). Hypoxia stress, which is caused by environmental deterioration in summer, can have lethal and sublethal consequences for harvested sea cucumbers, resulting in enormous mortality of the species in aquaculture and resource degradation. It has become one of the limiting factors for the sustainable development of the industry. However, despite several studies devoted to the physiological characteristics, including oxygen consumption rate, carbohydrate metabolism, and enzyme activity (Ross Ellington and Hammen 1977; Qian 2011; Zheng 2014; Li 2016), little is known about the molecular regulatory mechanisms that enable sea cucumbers to cope with hypoxia stress.

MicroRNAs (miRNAs) are endogenous noncoding small RNAs with 22 nucleotides, which play important roles in various physiological processes, including proliferation, differentiation, apoptosis, and immune responses (Bartel 2004; Chen *et al.* 2004; Croce and Calin 2005; Pedersen *et al.* 2007). By binding to target mRNA transcripts, miRNAs can reversibly inhibit translation of mRNAs and/or target them for degradation, thus they are now recognized as key regulators of gene expression (Bartel 2004). MiRNAs are sensitive to environmental changes, and their differential expression is important for the adaption of organisms to the environment (Kim *et al.* 2014). A previous study showed that miRNAs may play a key role in achieving a hypometabolic state among stress-tolerant animals (Chen *et al.* 2013). In recent years, a multitude of reports have demonstrated that specific miRNAs are involved in the hypoxic response and contribute to the regulation of hypoxia-related genes, such as *HIF*, *VEGF*, and *argonaute2* (Hua *et al.* 2006; Donker *et al.* 2007; Camps *et al.* 2008; Fasanaro *et al.* 2008; Guimbellot *et al.* 2009; Yeh *et al.* 2013). The regulation of organisms to cope with hypoxia stress is under the control of specific hypoxia-inducible miRs, which are also known as “hypoxamiRs” (Chan *et al.* 2012; Greco *et al.* 2014). MiR-210, the most accepted master hypoxamir (Chan *et al.* 2012), and other multiple hypoxamirs, including miR-200b (Ye *et al.* 2011), miR-199a (Rane *et al.* 2009), miR-21 (Cheng *et al.* 2010), miR-23a (Sayed *et al.* 2010), and miR-181c (Zhang *et al.* 2012), have been demonstrated to directly target important gene transcripts that regulate cell proliferation, DNA repair, antiinflammatory factors, apoptosis, and angiogenesis, among the adaptions in organisms toward hypoxic conditions. A few miRNA expression profiles have been successfully constructed in aquatic species under hypoxia stress, including river prawn (*Macrobrachium nipponense*), medaka (*Oryzias melastigma*), darkbarbel catfish (*Pelteobagrus vachelli*), and intertidal snail (*Littorina littorea*) (Biggar *et al.* 2012; Lau *et al.* 2014; Sun *et al.* 2016; Zhang *et al.* 2016). In *A. japonicus*, miRNA expression profiles constructed by high-throughput sequencing methods have been applied in different studies, such as regeneration, aestivation, skin ulceration syndrome (SUS), and specific tissues (Li *et al.* 2012; Chen *et al.* 2013; Wang *et al.* 2015; Sun *et al.* 2017). However, “hypoxamiRs” have not yet been reported in sea cucumber and very few have been reported in studies of Echinoderms. More efforts are needed to uncover the regulation mechanisms of miRNAs in response to hypoxia stress in Echinoderms.

In this work, we present for the first time an analysis of the global profile of small RNAs in sea cucumbers using Illumina sequencing technology, and compare them in hypoxia (DO 2 mg/L and DO 4 mg/L) and normoxic (DO 8 mg/L) states. We focus on the respiratory tree because it is the major tissue responsible for respiration and metabolism of sea cucumbers under adverse conditions. The main objectives are to identify and characterize miRNAs, which may play a major role in regulating genes related to hypoxia and stress resistance. Real-time PCR was used to confirm differentially expressed miRNAs. Our findings will be helpful in the further study of sea cucumber biomarkers and hypoxamiR identification under hypoxia stress and will provide important new insights into the molecular mechanisms in sea cucumbers for coping with hypoxic environments.

## Materials and Methods

### Animals

Sea cucumbers (body weight 100 ± 20 g) were collected from the coast of Weihai, China (East Ocean Science and Technology Co., Ltd). After weighing, they were acclimated in tanks containing aerated sand-filtered seawater (salinity 30‰, pH 8.0) at 15 ± 1° for 1 wk before use and were fed once a day at 11:00 am. Remaining feed was removed daily during the acclimation and experimental periods. All animals were then divided randomly into three groups. One group of sea cucumbers was maintained as the control group (DO8 group) in water with a high DO level of 8 mg/L, and the other two groups were kept in sea water with nitrogen aeration to gradually decrease DO to 4 mg/L (DO4 group) and 2 mg/L (DO2 group) for 3 d. Following exposure, specified quantities of sea cucumbers were dissected promptly and tissues were sampled to be preserved in liquid nitrogen and stored at −80° until subsequent analysis. In this study, three biological replicates were set in each group. The respiratory trees of three sea cucumbers were sampled for RNA extraction in each biological replicate. For instance, DO2_1, DO2_2, and DO2_3 were set in the DO2 group, which means that the nine healthy sea cucumbers were selected for further experimentation after exposure to DO levels of 2 mg/L for 3 d. No dead or dying animals were used in this section, ensuring the changes in miRNA expression were due to hypoxia.

### Small RNA library construction and sequencing

Respiratory tree tissue samples from nine sea cucumbers (three biological replicates × three sea cucumbers per biological replicate) randomly selected from each group were used in this study. Total RNA from each sample was extracted using an Animal Tissue RNA Purification Kit (LC Sciences, Houston, TX) according to the manufacturer’s instructions. Total RNA quality was checked with a Bioanalyzer 2100 (Agilent, Santa Clara, CA) with RNA integrity number >7.0. Total RNA of the sea cucumbers was mixed in equal amounts into nine pooled samples [severe hypoxia stress (DO 2 mg/L): DO2_1, DO2_2, DO2_3; mild hypoxia stress (DO 4 mg/L): DO4_1, DO4_2, DO4_3; normoxic condition (DO 8 mg/L): DO8_1, DO8_2, DO8_3]. Subsequently, a small RNA library was prepared according to the protocol of TruSeq Small RNA Sample Prep Kits (Illumina, San Diego, CA). Next, a 16–30 nt size range of RNA was purified from 15% polyacrylamide gels and then ligated sequentially to 5′ and 3′ adapters. Reverse transcription was performed followed by polymerase chain reaction (PCR) amplification. The purified PCR products were sequenced by Illumina Hiseq2500 Analysis (LC-BIO, Hangzhou, China).

### Sequence data analysis

The raw reads obtained from Hiseq2500 sequencing were trimmed. Clean data were processed by removing low-quality reads, reads with 5′ primer contaminants, reads without 3′ adapters, reads without insert fragments, reads of <18 nt, and reads containing poly(A). ribosomal RNA (rRNA), transfer RNA (tRNA), small nuclear RNA (snRNA) and small nucleolar RNA (snoRNA) were identified and removed by blasting against the GenBank database (http://blast.ncbi.nlm.nih.gov) and the Rfram database (http://rfam.xfam.org/). Because no miRNA information on the sea cucumber was in the miRBase21.0, the remaining clean reads were aligned to all known precursor/mature miRNAs of all animal species in miRBase 21.0 with ≤2 matches. The miRNAs with the highest expression for each mature miRNA family were selected as the temporary miRNA database. Clean data were aligned to the above temporary miRNA database, and the expression of miRNA was determined after summing the count of reads aligned to the temporary miRNA database with ≤2 mismatches. Finally, the precursor of the identified miRNAs was predicted, and molecules that could not fold into a hairpin structure were regarded as pseudo-miRNA. Potentially novel miRNAs were identified using MIREAP (http://sourceforge.net/projects/mireap/) with stem-loop structure prediction, as previously described (Chen *et al.* 2013).

Differentially expressed miRNAs were calculated by comparing the miRNA expression between control (DO8 group) and treatment (DO2 and DO4 group) samples. The expression of miRNA in control and treatment samples was normalized to determine the expression of reads per million (RPM). The normalization formula was: Normalized expression = Actual miRNA count/Total count of clean reads × 1,000,000. The final RPM between the biological replications was obtained through averaging. The log_2_(fold-change) and *P*-value were calculated from the normalized expression. Fold-change = Treatment-mean/Control-mean and *P*-value was:

$$P\left(y|x\right)={\left(\frac{N2}{N1}\right)}^{Y}\frac{\left(x+y\right)!}{x!y!{\left(1+\frac{N2}{N1}\right)}^{\left(X+Y+1\right)}}$$.

If the normalized expression was zero, it was changed to 0.01. A *P* < 0.05 was used as the criterion to determine the significance of the difference in miRNA expression. A heatmap of the differentially expressed miRNAs was constructed using the pheatmap package in R (version 3.1.3). All unique and shared differentially expressed miRNAs in the three groups were presented in a Venn diagram using Venny 2.1 (http://bioinfogp.cnb.csic.es/tools/venny/index.html).

### miRNA target prediction and gene ontology (GO) enrichment analysis

The 3′UTRs from the sea cucumber transcriptome assembly (Sun *et al.* 2011; Du *et al.* 2012) were extracted as the candidate database to predict the target genes using RNAhybrid software. Two computational target prediction algorithms (TargetScan 50 and miRanda 3.3a) were used to predict the genes targeted by differentially expressed miRNAs. The target genes were removed according to the following criteria: (1) context score percentile <50 calculated by TargetScan algorithms; and (2) max energy >−10 calculated by miRanda algorithms. The intersection of these two softwares was taken as the ultimate genes targeted by differentially expressed miRNAs. The network of predicted genes of miRNAs was shown using Cytoscape 2.8.3 software (Shannon *et al.* 2003).

The target genes of differentially expressed miRNAs were mapped to GO terms in the database (http://www.geneontology.org) using the program Blast2GO and the Kyoto Encyclopedia of Genes and Genomes Pathway database (KEGG) (http://www.genome.jp/kegg) for GO and KEGG analyses. The GO and pathway terms conforming to a *P* ≤ 0.05 through Bonferroni’s correction were defined as significantly enriched GO terms and pathways.

### Quantitative miRNA real-time PCR assay

For miRNA analyses, 1 μg of total RNA was reverse-transcribed with miRNA-specific stem-loop RT primers and reverse transcriptase M-MLV (RNase H^−^) (Takara, Shiga, Japan) according to the experimental protocol. Stem-loop reverse transcription primers were designed following a method described by Chen *et al.* (2005). The reaction proceeded for 10 min at 25°, 50 min at 42°, followed by 5 min at 85°, and a final hold at 4°. The cDNA was amplified by real-time PCR using Platinum SYBR Green qPCR SuperMix-UDG (Invitrogen, Carlsbad, CA) with miRNA-specific forward and reverse primers (Supplemental Material, Table S1) As an internal control to normalize for technical variations, 5.8 s rRNA was also amplified. All reactions were performed on three biological replicates, each of them being run three times. The 2^−ΔΔCT^ method was used to analyze the relative expression level of miRNA, and the level of significance was analyzed by SPSS statistics 18.0 software.

### Data availability

All raw data are available in the GEO database (GSE100603). Table S1 contains selected miRNAs and their primer sequences used for real-time PCR. Table S2 includes predicted target annotations of differentially expressed miRNAs. Figure S1 presents length distributions of reads in nine libraries.

## Results

### Small RNA library construction

**Figure 1:**
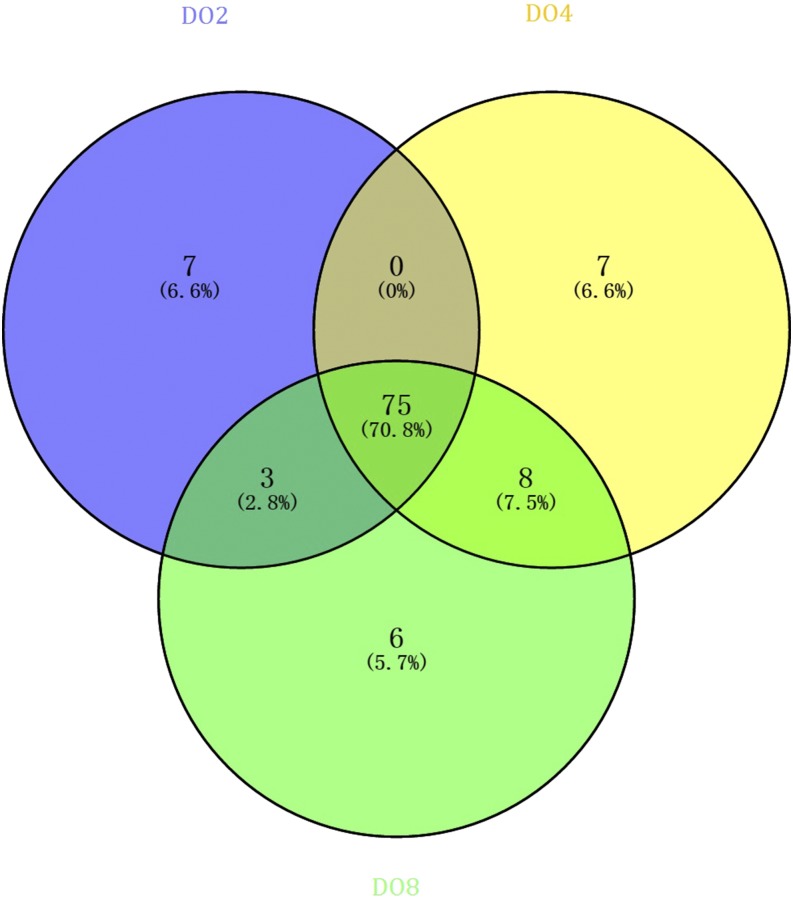
Common and specific sequence summary of unique miRNAs within the DO2 (DO 2 mg/L), DO4 (DO 4 mg/L), and DO8 (DO 8 mg/L) groups.

To identify differentially expressed miRNAs of sea cucumbers under hypoxia stress, nine small RNA libraries (treatments: DO2_1, DO2_2, and DO2_3; DO4_1, DO4_2, and DO4_3; controls: DO8_1, DO8_2, and DO8_3) were constructed from the respiratory tree of sea cucumber. All raw data have been submitted to the GEO database (GSE100603). High-throughput Illumina Hiseq2500 sequencing of these small RNA libraries yielded a total of 10,215,631 ± 689,938 (DO2), 10,901,071 ± 748,868 (DO4), and 11,064,987 ± 476,564 (DO8) high-quality clean reads, ranging from 16 to 30 nt after trimming the adapter sequences (Table 1). A total of 12,343 (DO2_1), 12,155 (DO2_2), 6413 (DO2_3), 1911 (DO4_1), 2930 (DO2_2), 3149 (DO2_3), 1442 (DO8_1), 1498 (DO8_2), and 3862 (DO8_3) unique small RNAs were identified as either rRNA (10,677, 10,094, and 4844 for DO2; 1566, 2396, and 2438 for DO4 ; 1055, 1032, and 3036 for DO8), snRNA (469, 511, and 373 for DO2; 48, 32, and 39 for DO4; 226, 19, and 28 for DO8), snoRNA (79, 102, and 91 for DO2; 35, 19, and 22 for DO4; 14, 14, and 24 for DO8), and tRNA (1107, 1363, and 926 for DO2; 303, 472, and 629 for DO4; 339, 429, and 766 for DO8) against NCBI GenBank and the Rfam 10.1 database using BLAST searches (Table 1). After removing the above small RNAs, the other RNAs were further analyzed for the identification of sea cucumber respiratory tree miRNAs against the latest miRBase release version 21.0. A total of 99, 180, and 299 unique miRNAs were searched in the three DO2 libraries; 142, 121, and 185 unique miRNAs were searched in the three DO4 libraries; and 103, 118, and 117 unique miRNAs were searched in the three DO8 libraries. The curves of the corresponding length distributions of reads show similar trends between these libraries by following a typical distribution pattern, and the majority of sequences were 22 nt in length (Figure S1). A Venn diagram shows the number of specific and common miRNAs in the three groups (Figure 1). Among them, 75 were classified as common miRNAs, seven as specific miRNAs in the DO2 library, seven as specific miRNAs in the DO4 library, and six as specific miRNAs in the DO8 library.

**Table 1:** Mapping statistics for sea cucumber RNA types from Illumina sequencing reads

	DO2_1				DO2_2				DO2_3			
lib	Total	% of Total	uniq	% of uniq	Total	% of Total	uniq	% of uniq	Total	% of Total	uniq	% of uniq
All clean reads	9,727,619	100.0000	560,047	100.0000	9,914,296	100.0000	404,613	100.0000	11,004,979	100.0000	143,712	100.0000
miRNA	539,420	5.5452	99	0.0177	1,063,953	10.7315	180	0.0445	2,841,858	25.8234	299	0.2081
rRNA	573,572	5.8963	10,677	1.9064	709,358	7.1549	10,094	2.4947	489,000	4.4434	4,844	3.3706
tRNA	31,558	0.3244	1,107	0.1977	44,265	0.4465	1,363	0.3369	51,574	0.4686	926	0.6443
snoRNA	1,146	0.0118	79	0.0141	1,219	0.0123	102	0.0252	1,572	0.0143	91	0.0633
snRNA	139,191	1.4309	469	0.0837	120,438	1.2148	511	0.1263	154,751	1.4062	373	0.2595
novel miRNA	89	0.0009	11	0.0020	536	0.0054	85	0.0210	3,164	0.0288	179	0.1246
Unann	8,442,643	86.7904	547,605	97.7784	7,974,527	80.4346	392,278	96.9514	7,463,060	67.8153	137,000	95.3295
	DO4_1				DO4_2				DO4_3			
lib	Total	% of Total	uniq	% of uniq	Total	% of Total	uniq	% of uniq	Total	% of Total	uniq	% of uniq
All clean reads	11,492,366	100.0000	221,088	100.0000	10,058,997	100.0000	157,861	100.0000	11,151,849	100.0000	100,967	100.0000
miRNA	10,541,644	91.7274	142	0.0642	8,404,678	83.5538	121	0.0766	9,326,745	83.6341	185	0.1832
rRNA	26,732	0.2326	1,566	0.7083	36,744	0.3653	2,396	1.5178	37,310	0.3346	2,438	2.4147
tRNA	6,474	0.0563	303	0.1370	7,643	0.0760	472	0.2990	7,608	0.0682	629	0.6230
snoRNA	327	0.0028	35	0.0158	533	0.0053	19	0.0120	202	0.0018	22	0.0218
snRNA	413	0.0036	48	0.0217	223	0.0022	32	0.0203	348	0.0031	39	0.0386
novel miRNA	797	0.0069	39	0.0176	235	0.0023	11	0.0070	168	0.0015	21	0.0208
Unann	915,979	7.9703	218,955	99.0352	1,608,941	15.9950	154,810	98.0673	1,779,468	15.9567	97,633	96.6979
	DO8_1				DO8_2				DO8_3			
lib	Total	% of Total	uniq	% of uniq	Total	% of Total	uniq	% of uniq	Total	% of Total	uniq	% of uniq
All clean reads	11,169,604	100.0000	156,487	100.0000	11,480,552	100.0000	134,370	100.0000	10,544,806	100.0000	180,413	100.0000
miRNA	10,182,919	91.1663	103	0.0658	9,740,816	84.8462	118	0.0878	8,056,432	76.4019	117	0.0649
rRNA	17,172	0.1537	1,055	0.6742	14,982	0.1305	1,032	0.7680	47,102	0.4467	3,036	1.6828
tRNA	5,624	0.0504	339	0.2166	6,673	0.0581	429	0.3193	10,780	0.1022	766	0.4246
snoRNA	457	0.0041	14	0.0089	308	0.0027	14	0.0104	454	0.0043	24	0.0133
snRNA	226	0.0020	31	0.0198	196	0.0017	19	0.0141	193	0.0018	28	0.0155
novel miRNA	58	0.0005	3	0.0019	55	0.0005	4	0.0030	74	0.0007	8	0.0044
Unann	963,148	8.6229	154,942	99.0127	1,717,522	14.9603	132,754	98.7974	2,429,771	23.0423	176,434	97.7945

Lib, Library; uniq, unique data; rRNA, ribosomal RNA; tRNA, transfer RNA; snoRNA, small nuclear RNA; snRNA, small nucleolar RNA; Unann, unannotated data.

### Different expression profiles of miRNAs and real-time PCR validation

In this study, 187 known miRNAs and 289 novel miRNAs were identified in the sea cucumber respiratory tree. The differentially expressed miRNAs were selected using a *t*-test with *P* < 0.05. Valid miRNAs were identified as those detected in all three groups from the same treatment condition, thus a total of 26 nonrepetitive miRNAs were identified to be differentially expressed in the pairwise comparison among the three treatments with significant levels (*P* < 0.05) (Table 2). Among the differentially expressed miRNAs, 12 upregulated miRNAs and 14 downregulated miRNAs were observed in severe hypoxia (DO2 group) compared with mild hypoxia (DO4 group) and normoxic conditions (DO8 group). Seven upregulated miRNAs and 12 downregulated miRNAs were obtained in severe hypoxia compared with normoxic conditions; three upregulated miRNAs and two downregulated miRNAs were obtained in mild hypoxia compared with normoxic conditions; six upregulated miRNAs and five downregulated miRNAs were obtained in the severe hypoxia compared with mild hypoxic conditions (Table 2). In addition, three novel miRNAs (novel-miR-1, novel-miR-2, and novel-miR-3) were identified. The log_2_Ratio $(\log_{2}\;\left(\frac{\text{DO2}\left(\text{mean}\right)}{\text{DO8}\left(\text{mean}\right)}\right)$ and $\log_{2}\;\left(\frac{\text{DO2}\left(\text{mean}\right)}{\text{DO4}\left(\text{mean}\right)}\right))$ of novel-miR-1 and novel-miR-2 and the log_2_Ratio $(\log_{2}\;\left(\frac{\text{DO2}\left(\text{mean}\right)}{\text{DO8}\left(\text{mean}\right)}\right)$ and$\;\log_{2}\left(\frac{\text{DO4}\left(\text{mean}\right)}{\text{DO8}\left(\text{mean}\right)}\right))$ of novel-miR-3 were >8, indicating that the three novel miRNAs were significantly upregulated under low DO conditions (2 mg/L and 4 mg/L). Moreover, Aja-miR-7977 and Aja-miR-2835 also meet the criterion of “absolute value of log_2_Ratio >8,” indicating that Aja-miR-7977 has significantly decreased expression and Aja-miR-2835 has significantly increased expression in severe hypoxia (DO 2 mg/L) (Table 2). Thus, they might be important to sea cucumber during hypoxia stress according to the analysis of high-throughput sequencing data.

**Table 2:** Differentially expressed miRNAs in sea cucumber respiratory tree among three different experiments (DO 2 mg/L, DO 4 mg/L, and DO 8 mg/L)

miR_name	DO2 (Mean ± SD)	DO4 (Mean ± SD)	DO8 (Mean ± SD)	log_2_ [DO2 (Mean)/DO8 (Mean)]	log_2_ [DO2 (Mean)/DO4 (Mean)]	log_2_ [DO4 (Mean)/DO8 (Mean)]
Aja-miR-2008	3,046 ± 368 (b)	833 ± 390 (a)	949 ± 69 (a)	1.6825	1.8710	−0.1885
Aja-miR-10-5p	908,819 ± 99,048 (a)	1,789,790 ± 217,900 (b)	1,614,760 ± 388,785 (ab)	−0.8293	−0.9777	0.1485
Aja-miR-184	25 ± 5 (b)	8 ± 5 (a)	10 ± 5 (a)	1.2841	1.6776	−0.3935
Aja-miR-71b	8 ± 8 (a)	34 ± 6 (b)	18 ± 5 (a)	−1.1502	−2.0310	0.8808
Aja-miR-125-5p	132,231 ± 32,089 (a)	355,285 ± 71,455 (b)	292,087 ± 14,330 (b)	−1.1433	−1.4259	0.2826
novel-miR-1	47 ± 11 (b)	0.01 ± 0 (a)	0.01 ± 0 (a)	12.1984	12.1984	0.0000
Aja-let-7a-5p	8 ± 9 (a)	35 ± 9 (b)	28 ± 5 (b)	−1.7813	−2.1054	0.3240
Aja-miR-375-3p	31,748 ± 5,645 (a)	15,970 ± 6,033 (b)	23,608 ± 8,389 (ab)	0.4274	0.9914	−0.5639
Aja-miR-2013-3p	606 ± 72 (a)	1,427 ± 292 (b)	1,331 ± 68 (b)	−1.1339	−1.2352	0.1012
novel-miR-2	33 ± 11 (b)	0.01 ± 0 (a)	0.01 ± 0 (a)	11.6883	11.6883	0.0000
Aja-miR-2835	54 ± 20 (b)	0.01 ± 0 (a)	0.01 ± 0 (a)	12.3987	12.3987	0.0000
Aja-miR-1	66 ± 24 (b)	9 ± 1 (ab)	7 ± 5 (a)	3.2340	2.9164	0.3175
Aja-miR-71-5p	58,756 ± 13,628 (a)	146,865 ± 44,428 (ab)	115,157 ± 12,697 (b)	−0.9708	−1.3217	0.3509
Aja-miR-200-3p	35,729 ± 3,207 (a)	95,032 ± 34,864 (ab)	102,503 ± 21,706 (b)	−1.5205	−1.4113	−0.1092
Aja-miR-2011-3p	49,272 ± 4,264 (a)	63,920 ± 17,541 (ab)	71,512 ± 1,331 (b)	−0.5374	−0.3755	−0.1619
Aja-miR-2478a	30 ± 23 (a)	124 ± 117 (ab)	151 ± 37 (b)	−2.3305	−2.0527	−0.2778
Aja-miR-31-5p	9,141 ± 418 (a)	9,179 ± 4,431 (ab)	11,421 ± 819 (b)	−0.3212	−0.0059	−0.3153
Aja-miR-7977	0.01 ± 0 (a)	4 ± 5 (ab)	6 ± 2 (b)	−9.2288	−8.6439	−0.6406
Aja-miR-71a	1,792 ± 345 (a)	3,346 ± 3,226 (ab)	2,688 ± 346 (b)	−0.5848	−0.9008	0.3160
Aja-miR-29b-3p	3,179 ± 241 (b)	2,828 ± 799 (ab)	2,400 ± 345 (a)	0.4055	0.1690	0.2365
Aja-miR-2478b	0.01 ± 0 (a)	1 ± 2 (ab)	2 ± 1 (b)	−7.6439	−6.6439	−0.8295
Aja-miR-2008-5p	88 ± 34 (a)	171 ± 114 (ab)	190 ± 48 (b)	−1.1100	−0.9636	−0.1464
Aja-miR-1a-3p	101,709 ± 26,157 (ab)	77,382 ± 10,825 (b)	55,209 ± 7,288 (a)	0.8815	0.3944	0.4871
novel-miR-3	27 ± 24 (ab)	5 ± 1 (a)	0.01 ± 0 (a)	11.3987	2.5481	8.9658
Aja-miR-153-3p	178 ± 106 (ab)	39 ± 7 (a)	57 ± 3 (b)	1.6360	2.1911	−0.5551
Aja-miR-153	23 ± 17 (ab)	0.01 ± 0 (a)	4 ± 1 (b)	2.6895	3.3617	−8.6439

Values indicate the means ± SD (n = 3). Means not sharing a given letter (a and b) differ significantly (P < 0.05).

**Figure 2:**
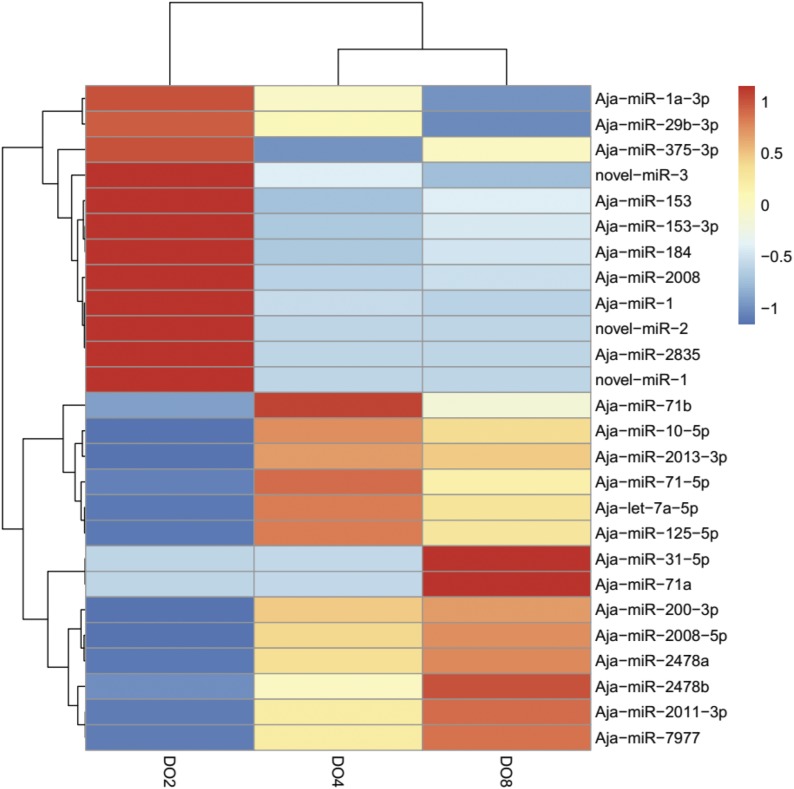
Heatmap of differentially expressed miRNAs in three different experiments (DO 2 mg/L, DO 4 mg/L, and DO 8 mg/L) by hierarchical clustering. Red indicates higher levels of miRNAs and blue indicates lower levels of miRNAs.

The heatmap of 26 differentially expressed miRNAs (*P* < 0.05) in three experiment groups are illustrated in Figure 2. As Figure 2 shows, 12 of the miRNAs are clustered in severe hypoxic conditions (DO2 group), including novel-miR-1, novel-miR-2, novel-miR-3, Aja-miR-1, and Aja-miR-2008; six of them are clustered in mild hypoxic conditions (DO4 group), including Aja-miR-71b, Aja-miR-71-5p, and Aja-miR-125-5p; eight of them are clustered in normoxic conditions (DO8 group), including Aja-miR-2011-3p, Aja-miR-31-5p, and Aja-miR-200-3p. We speculated that some of them may be crucial for regulating hypoxia-associated gene expression and play important roles in stress resistance. Notably, the three novel miRNAs (novel-miR-1, novel-miR-2, and novel-miR-3) identified by this study were all clustered in the severe hypoxic condition (Figure 2), which may provide new insights into novel hypoxia biomarker identification.

**Figure 3:**
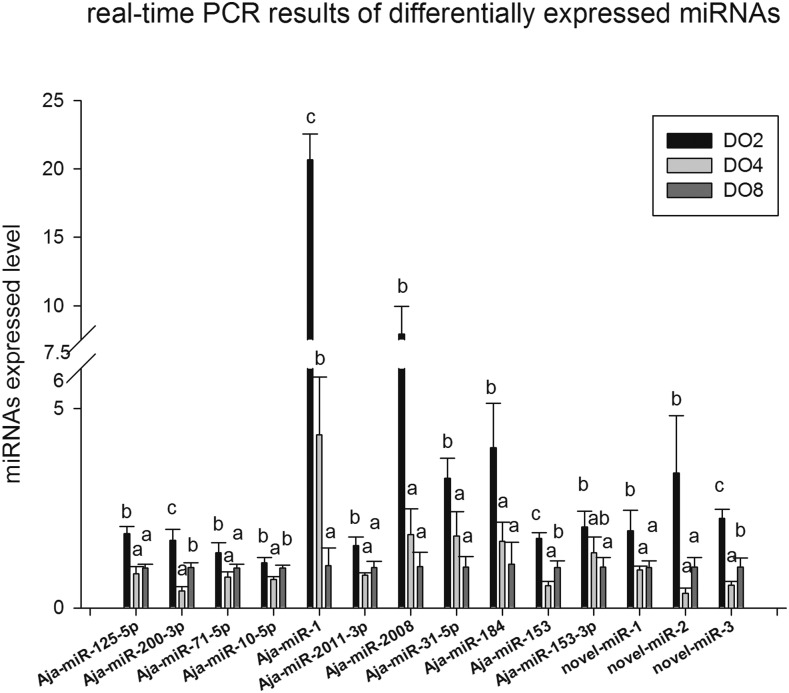
Real-time PCR analyses for 14 differentially expressed miRNAs identified by high-throughput sequencing. Means not sharing a given letter (a and b) differ significantly (*P* < 0.05). Values indicate the means ± SE (*n* = 3).

For the validation and identification of the hypoxia-related miRNAs in the sea cucumber, 14 differentially expressed miRNAs (Aja-miR-1, Aja-miR-184, novel-miR-1, novel-miR-2, novel-miR-3, Aja-miR-125-5p, Aja-miR-200-3p, Aja-miR-71-5p, Aja-miR-10-5p, Aja-miR-2011-3p, Aja-miR-2008, Aja-miR-31-5p, Aja-miR-153, and Aja-miR-153-3p) identified by high-throughput sequencing in hypoxia treatment were validated using real-time PCR (Figure 3). Real-time PCR results showed that the expression of the selected 14 miRNAs was significantly upregulated in the DO2 group compared with the DO4 and DO8 groups. Notably, Aja-miR-1 was upregulated 19.5-fold; Aja-miR-2008 was upregulated 7.6-fold; Aja-miR-184 was upregulated 3.7-fold, novel-miR-2 was upregulated 7.6-fold, and Aja-miR-31-5p was upregulated 3.2-fold in severe hypoxic compared with normoxic conditions. The expressions of nine miRNAs (Aja-miR-200-3p, Aja-miR-1, Aja-miR-2008, Aja-miR-184, Aja-miR-153, Aja-miR-153-3p, novel-miR-1, novel-miR-2, and novel-miR-3) were consistent with the overall trend in Illumina sequencing. Among these molecules, the expression of Aja-miR-1, Aja-miR-2008, Aja-miR-31-5p, Aja-miR-184, and Aja-miR-153-3p were different under three different levels of dissolved oxygen with a dose-response effect, which means the expression increased with the decreased content of dissolved oxygen. The different expression of Aja-miR-1, Aja-miR-200-3p, Aja-miR-153, and novel-miR-3 was extremely significant in three experiment groups validated by real-time PCR.

### GO and pathway enrichment analysis for target genes of the miRNAs

**Figure 4:**
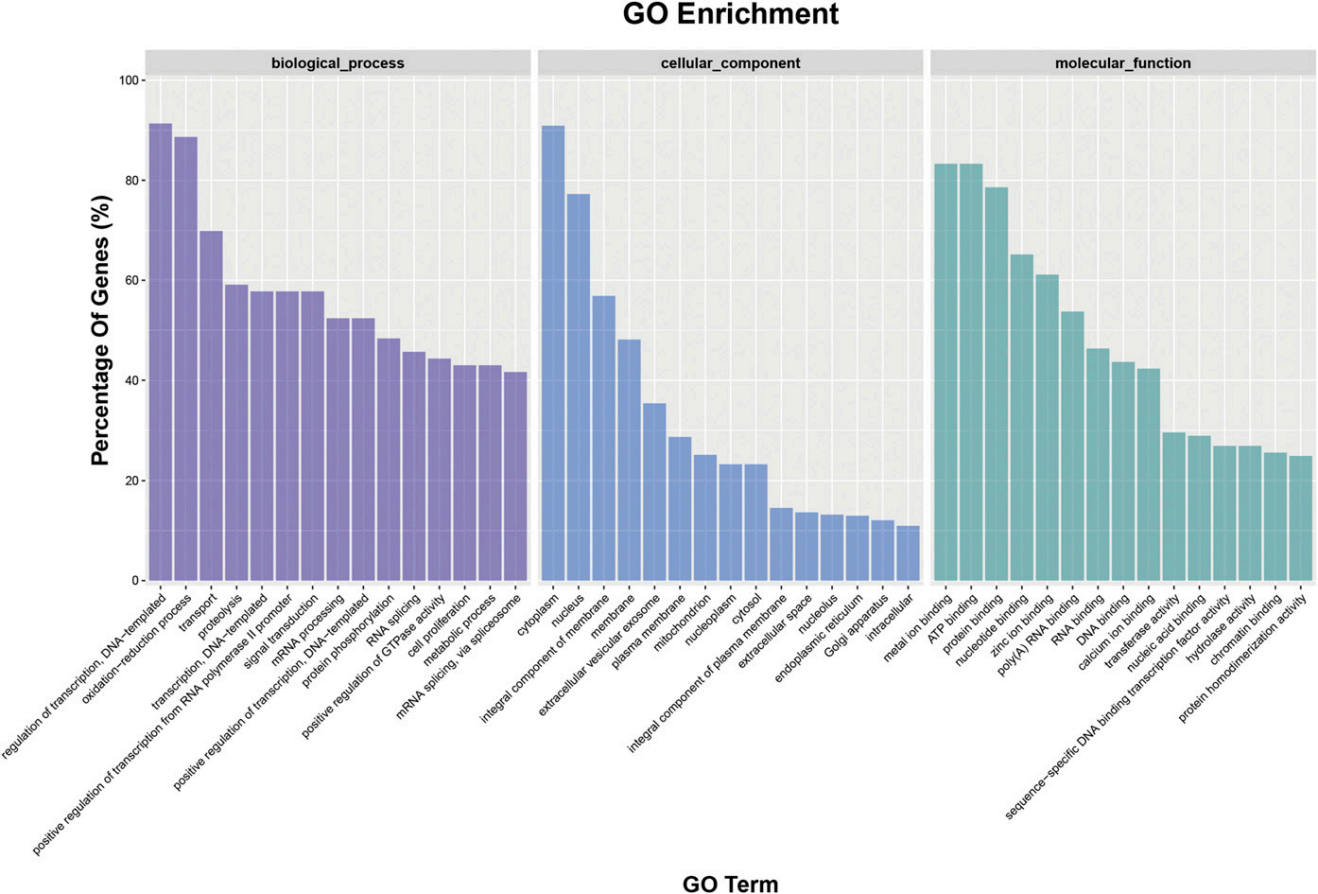
GO enrichment terms of the predicted target genes of differentially expressed miRNAs (*P* < 0.05).

**Figure 5:**
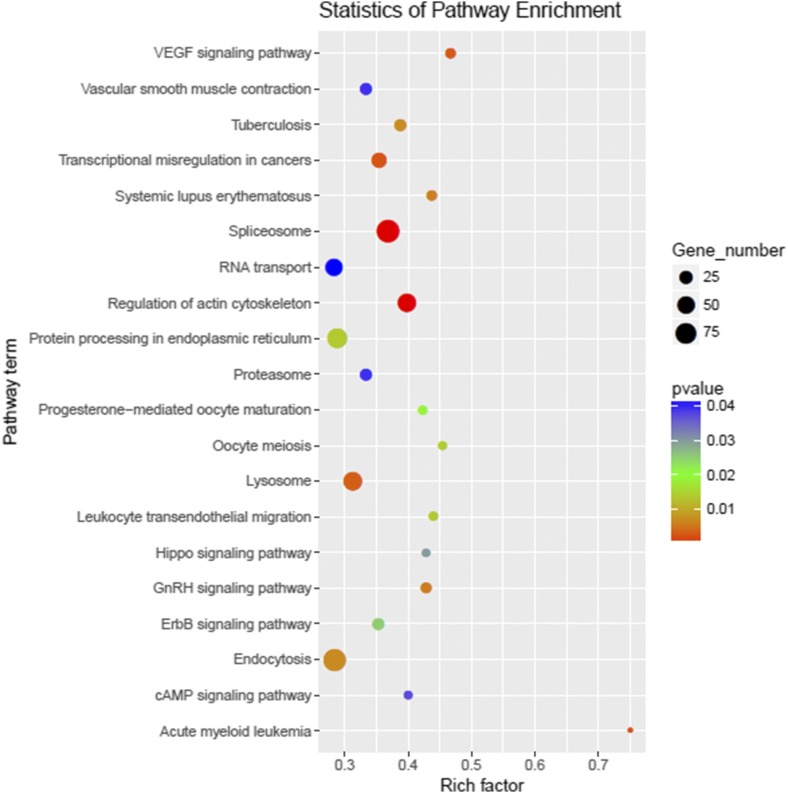
Pathway enrichment of the predicted target genes of differentially expressed miRNAs.

To further understand the biological function of miRNAs, the putative target genes of differentially expressed miRNAs were predicted and the annotations are shown in Table S2. Subsequently, GO analysis was used to identify enriched functional groups (*P* < 0.05) (Figure 4). These target genes predominantly participate in biological processes, cellular component and molecular function. Within the terms of biological processes, the three most frequent categories were “regulation of transcription,” “oxidation-reduction processes,” and “transport.” The three most highly represented molecular component categories were “cytoplasm,” “nucleus,” and “integral component of membrane.” Finally, the three most abundant molecular function categories were “metal ion binding,” “ATP binding,” and “protein binding.” Enriched metabolic pathways and signal transduction pathways were identified and are listed in Figure 5. Twenty-six significantly enriched pathways for target genes (*P* < 0.05) mainly involved in “Spliceosome,” “Regulation of actin cytoskeleton,” “Acute myeloid leukemia,” “Transcriptional misregulation in cancers,” “VEGF signaling pathway,” “Lysosome,” “GnRH signaling pathway,” “Systemic lupus erythematosus,” “Endocytosis,” and “Tuberculosis” were screened (Figure 5).

## Discussion

The miRNA expression profiles of the respiratory tree in sea cucumber under different DO levels and obtained using high-throughput sequencing technology are reported in this study. A total of nine libraries were constructed, and ∼90 million reads were obtained. These results will not only play a complementary role in improving the amount of information for the small RNA genome of the sea cucumber but also provide the basis for miRNA regulation in stress resistance and hypoxamiR identification. In our study, 19 miRNAs were significantly differentially expressed between the comparison of severe hypoxia and normoxic conditions (DO2 *vs.* DO8 group); five between mild hypoxia and normoxic conditions (DO4 *vs.* DO8 group); and 11 between severe hypoxia and mild hypoxia (DO2 *vs.* DO4 group). The hierarchical clustering result of differentially expressed miRNAs showed that 12 were clustered in the DO2 group, six were clustered in the DO4 group, and eight were clustered in the DO8 group. Real-time PCR was used to verify the expression profile. Fourteen miRNAs were significantly differentially expressed, most of which have been demonstrated to play an important role in oxidative response, apoptosis, cell proliferation, and migration (Nohata *et al.* 2011; Boulias and Horvitz 2012; Jiang *et al.* 2016). Combined with the published conclusions in previous studies (Liu *et al*, 2010a; Liu *et al*, 2010b; Liu *et al*, 2015; Nohata *et al*, 2011; Li *et al*, 2012), sequencing results, and validation results of expression levels, we speculated that key miRNAs, including Aja-miR-1, Aja-miR-2008, Aja-miR-184, and Aja-miR-31-5p, may play important roles in regulating hypoxia-associated gene expression, thereby enhancing the ability of stress resistance. This result was expected, as a complicated process is involved in the adaption to hypoxia stress.

**Figure 6:**
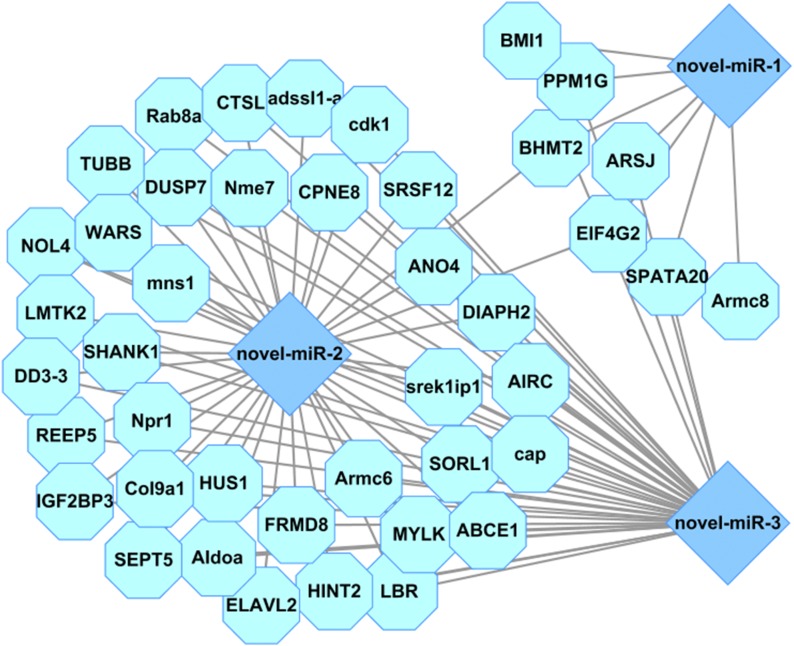
Predicted target genes of three novel miRNAs (novel-miR-1, novel-miR-2, and novel-miR-3).

Three novel miRNAs (novel-miR-1, novel-miR-2, and novel-miR-3) were identified in this study and were specifically clustered in the DO2 group as analyzed by hierarchical clustering (Figure 2). The real-time PCR results showed that novel-miR-1 and novel-miR-3 were both upregulated about twofold and novel-miR-2 was upregulated about threefold under severe hypoxia (DO 2 mg/L) compared with normoxic conditions (DO 8 mg/L) and were consistent with sequencing. Thus, we speculated that they may be important in sea cucumber stress response and adaption under hypoxia and they might be identified as new hypoxamiRs. The key predicted genes of these three novel miRNAs are shown in Figure 6. Cathepsin-L (CTSL) was the predicted gene of novel-miR-2 and novel-miR-3. It has been reported that the production of CTSL was induced in fibroblasts and KHT-LP1 cells exposed to hypoxia (Anderson *et al.* 1989; Cuvier *et al.* 1997). Furthermore, CTSL was able to broadly affect the immune system and might be involved in increased invasion capacity (Cuvier *et al.* 1997; Lombadi *et al.* 2005). In addition, fructose-bisphosphate aldolase A (ALDOA) was also the copredicted gene of novel-miR-2 and novel-miR-3. ALDOA, a type of glycolytic enzyme which could convert glucose to lactate, was identified to be upregulated in hypoxic conditions and could be mediated by hypoxia-inducible factor 1 (HIF-1) (Sørensen *et al.* 2009; Semenza 2012; Leisz *et al.* 2015). HIF-1 is reported to play important roles in molecular adaption to hypoxia and is primarily responsible for the subsequent activation of several other hypoxia-responsive genes involved in glycolysis, erythropoiesis, catecholamine metabolism, angiogenesis, transposons and iron metabolism, growth suppressor genes, and several other genes directly linked to the absence of oxygen (Bracken *et al.* 2003; Egg *et al.* 2013; Semenza 2014). Thus, we analyzed the potential targeted relationship between novel-miR-2 and HIF-1 in sea cucumber by a manual blast search of the sequence. Results showed that novel-miR-2 was predicted to match 6 bp with the seed sequence of HIF-1α in *A. japonicus*. Furthermore, ALDOA was associated with hypoxia and the antioxidant stress response and has a positive role in ROS production (Ji *et al.* 2016). Moreover, Armadillo repeat-containing protein 8 (Armc8), the predicted gene of novel-miR-1, was reported to play an important role in regulating cell migration, proliferation, tissue maintenance, and signal transduction (Jiang *et al.* 2016). Armc8 is also a key component of the CTLH (C-terminal to lissencephaly type-1-like homology motif) complex of mammalian cells, which has been characterized as an FBPase (fructose-1, 6-bisphosphatase)-degrading complex (Zhao *et al.* 2016). In conclusion, the three novel miRNAs may play important roles in the response and adaption of sea cucumbers under hypoxia stress by mediating key genes related to hypoxia such as HIF-1 and genes involved in glycolysis, cell migration, and proliferation.

Aja-miR-2008 was clustered in the DO2 group when analyzed by hierarchical clustering (Figure 2), and it was upregulated ∼7.6-fold in severe hypoxia compared with normoxic conditions as validated by real-time PCR, and the result was consistent with sequencing. MiR-2008 was previously found to be significantly upregulated in diseased sea cucumber by high-throughput sequencing (Zhang *et al.* 2015). In previous studies, miR-2008 was significantly upregulated after pathogen infection and was speculated to be involved in a SUS outbreak (Li *et al.* 2012; Zhang *et al.* 2014a). Under hypoxia stress in this study, several sea cucumbers were observed to be distorted with ulcerated skin, the papillae of the sea cucumbers became white during the early stage of hypoxia stress, and then the body walls dissolved. These symptoms are similar to SUS; therefore the upregulated expression of Aja-miR-2008 was not surprising.

According to the miRNA profile of sea cucumbers in this study, the miR-71 family showed a different expression level under hypoxia stress. The expression of Aja-miR-71-5p was upregulated ∼1.4-fold under severe hypoxia compared with normoxic conditions as validated by real-time PCR and this tendency was in accordance with sequencing, and the expression levels of Aja-miR-71a and Aja-miR-71b were downregulated. MicroRNA-71 (miR-71) can increase resistance to heat shock and oxidative stress (Boulias and Horvitz 2012). Moreover, miR-71 has previously been linked with the process of aging and the DNA damage response pathway (De Lencastre *et al.* 2010). In addition, a study associated miR-71 with a response to environmental stresses and nutrient availability through interactions with different targets of the insulin and PI-3K signaling pathways in *Caenorhabditis elegans* (Zhang *et al.* 2011). Therefore, we speculated that the miR-71 family may play important roles in regulating the hypoxia stress response by targeting genes related to stress resistance.

Aja-miR-31-5p was upregulated ∼3.2-fold under severe hypoxia (DO2 group) compared with normoxic conditions (DO8 group) by real-time PCR. Former studies showed that miR-31 is involved in several signaling pathways, which can mediate cell proliferation, apoptosis, and DNA mismatch repair. For example, miR-31 could increase cell migration, invasion, and proliferation in an ERK1/2 signaling-dependent manner (Meng *et al.* 2013). Moreover, miR-31-5p has an important role in radiation responses through regulation of hMLH1 expression, which is one of the core DNA mismatch repair genes that also decreases expression under hypoxia stress (Mihaylova *et al.* 2003; Kim *et al.* 2014). MiR-31 was also speculated to be involved in a SUS outbreak in a similar way to miR-2008 (Li *et al.* 2012). Above all, the research suggested that miR-31-5p may play an important role in hypoxia-related genes such as *EGFR*, *VEGF*, and *HIF*. MiR-31-5p could regulate the signaling pathway downstream of EGFR, which has been shown to induce HIF-1α expression in previous studies (Pore *et al.* 2006; Igarashi *et al.* 2015). Furthermore, a previous study suggested that miR-31 contributes to the development of head and neck squamous cell carcinoma by impeding factor inhibiting HIF (FIH) to activate HIF under normoxic conditions (Liu *et al.* 2010b). Thus, Aja-miR-31-5p could play an important role through the EGFR/HIF-1α/VEGF pathways in stress resistance under hypoxic conditions.

Aja-miR-184 was upregulated when analyzed by sequencing and specifically clustered into the DO2 group. It was upregulated ∼3.7-fold under severe hypoxia when validated by real-time PCR. MiR-184 can cause apoptosis when overexpressed and increase cell numbers when inhibited (Foley *et al.* 2010). Previous studies have shown that miR-184 overexpression inhibits autophagy and exacerbates oxidative damage (Liu *et al.* 2015). Reports by others provide strong evidence to support the idea that miR-184 is an important modulator of stem cell proliferation and growth (Liu *et al.* 2010a). Furthermore, miR-184 is reported to be involved in many pathways and some of them are related to the oxidative response. For example, miR-184 negatively modulates Wnt signaling both *in vivo* and *in vitro* (Takahashi *et al.* 2015). MiR-184 could inhibit protein expression in human trabecular meshwork cell cytotoxicity, apoptosis, and extracellular matrix via targeting HIF-1α *in vivo*, and it can also exhibit angiostatic properties through regulating signaling pathways including Akt, TNF-α, and VEGF (Park *et al.* 2017; Wang *et al.* 2017). All the results mentioned above showed that Aja-miR-184 may be crucial to the stress response in sea cucumbers by regulating genes related to autophagy, oxidative damage, and cell proliferation under hypoxia stress.

Aja-miR-1 was highly expressed by ∼19.5-fold under severe hypoxia when analyzed by real-time PCR and the tendency was consistent with high-throughput sequencing results. MiR-1 has been demonstrated to be associated with apoptosis-related genes such as heat shock protein (HSP), and indirectly regulates eNOs (He *et al.* 2011). A former study indicated that miR-1 regulated Hsp60 expression post-transcriptionally and accelerated cardiomyocyte apoptosis through Hsp60 (Shan *et al.* 2010). Moreover, apoptosis are mediated by miR-1 (Yu *et al.* 2008). Restoration of miR-1 in cancer cells inhibits cell proliferation, invasion, and migration (Nohata *et al.* 2011). Previous results indicate that miR-1 directly regulates the levels of VegfA (Vascular endothelial growth factor A) in muscle, which is regulated by the HIF gene in hypoxia (Stahlhut *et al.* 2012). MiR-1 levels were significantly increased in response to oxidative stress (Tang *et al.* 2009). The results reported in previous studies are consistent with the present research.

In addition to the results of high-throughput sequencing, real-time PCR validation and hierarchical clustering, Aja-miR-2835, Aja-miR-153, and Aja-miR-153-3p are also found to be key miRNAs, which may be important in regulating hypoxia-associated gene expression. Although their physiological functions remain unclear, their expression patterns and target genes indicate that these miRNAs are likely to play important roles in hypoxia stress tolerance. Further experiments are needed to elucidate the roles of these molecules in this process.

To obtain insight into the potential function of differentially expressed miRNAs under hypoxia stress, we performed a GO and pathway enrichment analysis of their predicted targets. The significantly enriched GO terms were “regulation of transcription,” “oxidation-reduction processes,” “transport,” “proteolysis,” “transcription,” and “positive regulation of transcription,” which are primarily associated with redox, transport, transcription, and hydrolysis. This can be partially explained by the complex biological process involved in sea cucumbers under hypoxia stress, which includes material transport, oxidative stress, and transcription regulation. By searching KEGG, a total of 26 significantly enriched pathways were observed (*P* < 0.05), with the top 10 significant pathways being “Spliceosome,” “Regulation of actin cytoskeleton,” “Acute myeloid leukemia,” “Transcriptional misregulation in cancers,” “VEGF signaling pathway,” “Lysosome,” “GnRH signaling pathway,” “Systemic lupus erythematosus,” “Endocytosis,” and “Tuberculosis.” Since HIF-1α, a typical hypoxia biomarker reported in other species, is known to be related to hypoxia stress and involved in the “VEGF signaling pathway” (Zhang *et al.* 2014b), and the differentially expressed miRNA, miR-1, was reported to directly regulate the expression of *VEGF* (Stahlhut *et al.* 2012), it was not surprising that this pathway was enriched. In addition, hypoxia stress is supposed to be related to immunity, so some pathways related to immune response were also identified, such as “Lysosome,” “Spliceosome,” and “Endocytosis” (Figure 5).

In conclusion, this study provides a global view of miRNA changes under hypoxia stress. A total of 26 nonrepetitive miRNAs were significantly differentially expressed in three levels of dissolved oxygen, and these molecules were considered to be responsible for the stress resistance of sea cucumbers. The key miRNAs, analyzed by high-throughput sequencing, real-time PCR validation, and hierarchical clustering, such as Aja-miR-1, Aja-miR-2008, Aja-miR-184, and Aja-miR-31-5p, might be significant in regulating hypoxia-associated gene expression. These miRNAs may play important roles in redox, transport, transcription, hydrolysis, and tolerance ability under hypoxic stress, indicating that sea cucumbers cope with hypoxic stress using a complex biological process, which includes material transport, oxidative stress, and transcription regulation. Moreover, the three novel miRNAs (novel-miR-1, novel-miR-2, and novel-miR-3) identified in the present study were all clustered and significantly upregulated in severe hypoxia, which may provide the basis for hypoxamiR research and novel hypoxia biomarker identification. These results provide an insight into the molecular mechanisms that occur in sea cucumber under hypoxia stress.

## Supplementary Material

Supplemental material is available online at www.g3journal.org/lookup/suppl/doi:10.1534/g3.117.1129/-/DC1.
