# Supplementary figures and images for: Differential Expression of miRNAs in the Respiratory Tree of the Sea Cucumber Apostichopus japonicus Under Hypoxia Stress
Source: G3 (Bethesda). 2017 Sep 15;7(11):3681–92. doi: 10.1534/g3.117.1129 (PMC5677170; doi:10.1534/g3.117.1129)

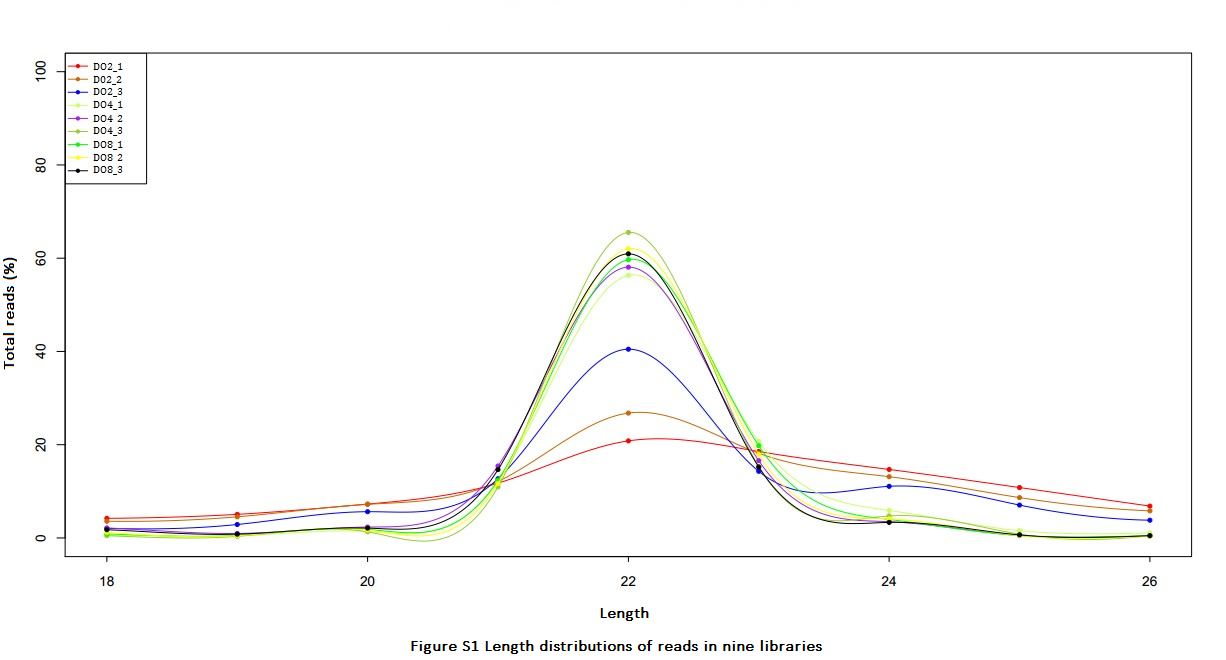

Supplement: Supplementary file 1 [file 3681FigureS1.tif]
