# Supplementary material for: Differential Expression of miRNAs in the Respiratory Tree of the Sea Cucumber Apostichopus japonicus Under Hypoxia Stress
Source: G3 (Bethesda). 2017 Sep 15;7(11):3681–92. doi: 10.1534/g3.117.1129 (PMC5677170; doi:10.1534/g3.117.1129)
Supplement: Supplementary file 2 [file 3681TableS1.pdf]

Table S1 Selected miRNAs and their primer sequences used for real-time PCR

MiRNAs name	Primer sequence (5'to3')
Aja-miR-2013-3p	R: GCGCGT GAGCAGGCTGGAGAA ATTAACCA CGCGC AACACC
	F: CGTGCAGCATGATGTAG
Aja-miR-125-5p	R: GCGCGT GAGCAGGCTGGAGAA ATTAACCA CGCGC TCACAA
	F: GTCCCTGAGACCCTAAC
Aja-miR-200-3p	R: GCGCGT GAGCAGGCTGGAGAA ATTAACCA CGCGC CATCATC
	F: CGGTAATACTGTCTGGTG
Aja-miR-71-5p	R: GCGCGT GAGCAGGCTGGAGAA ATTAACCA CGCGC TCTCAC
	F: GCTGAAAGACATGGGTAG
Aja-miR-10	R: GCGCGT GAGCAGGCTGGAGAA ATTAACCA CGCGC ACAAAT
	F: CGTACCCTGTAGATCCG
Aja-miR-1	R: GCGCGT GAGCAGGCTGGAGAA ATTAACCA CGCGC ATACTTC
	F: GCGGTGGAATGTAAAG
Aja-miR-2008	R: GCGCGT GAGCAGGCTGGAGAA ATTAACCA CGCGC TCGTAT
	F: TCAGCCTCGCTGTCAA
Aja-miR-31-5p	R: GCGCGTGAGCAGGCTGGAGAA ATTAACCA CGCGC AGCTATG
	F: TAGGCAAGATGTTGGC
Aja-miR-184	R: GCGCGT GAGCAGGCTGGAGAA ATTAACCA CGCGC ACTTATC
	F: GGTGGACGGAGAACTG
Aja-miR-153	R: GCGCGT GAGCAGGCTGGAGAA ATTAACCA CGCGC CTGTCA
	F: GGCGTCTCTCTCTCTCTG
Aja-miR-153-3p	R: GCGCGT GAGCAGGCTGGAGAA ATTAACCA CGCGC AATCAC
	F: AGTTGCATAGTCACAAAAG
novel-miR-1	R: GCGCGT GAGCAGGCTGGAGAA ATTAACCA CGCGC TCAACA
	F: GTGGCACGTGACTTTG
novel-miR-2	R: GCGCGT GAGCAGGCTGGAGAA ATTAACCA CGCGC GGATCTG
	F: CCAAAGAAAGCCTCTAAC
novel-miR-3	R: GCGCGT GAGCAGGCTGGAGAA ATTAACCA CGCGC TTTCAG
	F: ATCAAAGGTTGAGGGC
Aja-cytochrome b (CYTB)	R: AAAAGGGAAAAGGAAGTGGAAG
	F: TTTCTGAGCCGCAACAGTAATC
universal R primer	GAGCAGGCTGGAGAA
